# Mapping for Conceptual Clarity: Exploring Implementation of Integrated Community-Based Primary Health Care from a Whole Systems Perspective

**DOI:** 10.5334/ijic.3082

**Published:** 2018-03-21

**Authors:** Carolyn Steele Gray, Walter P. Wodchis, G. Ross Baker, Peter Carswell, Tim Kenealy, Ann McKillop, Mylaine Breton, John Parsons, Nicolette Sheridan

**Affiliations:** 1 Bridgepoint Collaboratory for Research and Innovation, Lunenfeld-Tanenbaum Research Institute, Sinai Health System, CA; 2 Institute of Health Policy, Management and Evaluation, University of Toronto, CA; 3 Research Chair in Implementation and Evaluation Science, Institute for Better Health, Trillium Health Partners, CA; 4 School of Population Health, The University of Auckland, NZ; 5 South Auckland Clinical School, The University of Auckland, NZ; 6 School of Nursing, Faculty of Medical and Health Services, The University of Auckland, NZ; 7 Charles LeMoyne Hospital Research Center, Université de Sherbrooke, Quebec, CA; 8 School of Nursing, College of Health *Te Kura Haurora Tengata*, Massey University, NZ

**Keywords:** Concept map, integrated care, primary health care, methods, whole systems, complex care needs

## Abstract

**Introduction:**

Studying implementation of integrated models of community-based primary health care requires a “whole systems” multidisciplinary approach to capture micro, meso and macro factors. However, there is, as yet, no clear operationalization of a “whole systems” approach to guide multidisciplinary research programs. Theoretical frameworks and approaches from diverse academic traditions specify different aspects of the health system in more depth. Enabling analysis across the system, when data and ideas are captured using different taxonomies, requires that we map terms and constructs across the models.

**Theory and methods:**

This paper uses concept mapping techniques to compare and contrast the theoretical frameworks and approaches used in the iCOACH project including: Ham’s Ten Characteristics of the High-Performing Chronic Care System (capturing patient/carer and provider perspectives), the Organizational Context and Capabilities for Integrating Care framework (capturing the organizational perspective), and the Health Policy Monitor framework (capturing the policy system perspective). The aim of the paper is to link concepts across different theoretical framework to guide the iCOACH study.

**Results:**

A concept map was developed that identifies 8 overarching concepts across the heuristic models. A preliminary analysis of one of these overarching concepts, care coordination, demonstrates how different perspectives will assign different meanings, values, and drivers of seemingly similar ideas. For patients and carers care coordination is about having a responsive team of health care providers. Building relationships in teams that exist within and across different organizations is essential for providers to achieve care coordination, where managers and policy makers see care coordination as being more about creating linkages and addressing systems gaps.

**Discussion and conclusion:**

This work represents a first step towards development of a fully formed conceptual framework that includes key domains, concepts, and mechanisms of implementing integrated community-based primary health care.

## Introduction

Wagner’s Chronic Care Model (CCM) [1] (extended in 2003) [2] has been used as a best practice model in the delivery of care to patients with chronic and complex conditions. The CCM provides guidance on what elements should be adopted to improve chronic care management in practice with the CCM being particularly focused on primary care [3]; paying attention to factors at micro (patient/carer and provider), meso (organization) and macro (system/policy) levels. While outcomes related to CCM [4] and implementation of CCM [3] have been explored, there is limited understanding of factors facilitating the adoption of the CCM in ***integrated community-based primary health care (ICBPHC) settings*** to appropriately care for patients with complex care needs. With their multiple co-occurring chronic illnesses and social complexity [5], this patient population is most in need of a patient-centred approach to chronic disease management [6,7], however strategies to address multi-morbidity have mainly focused on formal health service provision through general practice and secondary care that are often fragmented with poor coordination of care [8,9,10,11]. Linking together multiple episodes of care offered by providers across the health system (such as the services identified in the CCM) has been identified as central to supporting patient-centredness [7]. Furthermore, core components of patient-centredness, such as integration of medical and non-medical care, coordination and continuity [12] are supported through integrated models.

In order to better understand the implementation of innovative models of ICBPHC, the *Implementing Integrated Models of Primary Health Care for Older Adults with Complex Needs (iCOACH)* research team takes a comprehensive “whole systems” approach, incorporating a policy analysis, organizational, provider and patient/carer level perspectives on implementing and delivering integrated care. “Whole systems” approaches recognize that relationships between different hierarchical levels will impact on effectiveness of the overall health system [13], and have been identified as essential to support our understanding of implementation of new models of care [14]. Each level of analysis is led by one of four research teams who have developed robust conceptualizations of pertinent issues relevant to their level of analysis and disciplinary approach. Three theoretical frameworks are brought to bear on this research question including: 1) Ham’s Ten Characteristics of the High-Performing Chronic Care System [15] (which captures key elements of the CCM) to capture patient/carer and provider level perspectives; 2) Organizational Context and Capabilities for Integrating Care (CCIC) framework [16] to capture organizational and provider level perspectives; and 3) the Health Policy Monitor (HPM) [17] survey methodology to capture system and policy level perspectives. See Wodchis et al. [18] and Breton et al. [19] in this issue for an overview of the research program and case study summaries.

The challenge, however, was in the identification of how frameworks used in parallel to study the same cases of ICBPHC overlapped. What is required is conceptual linkage when looking across different theoretical frameworks and disciplinary approaches. Conceptual linkage, we argue, is essential in studies of complex interventions like models integrated care which often rely on multiple *mechanisms of action* that may relate to different theories and disciplinary perspectives [20]. It is also particularly important for studies engaged in “whole systems” health services research, which requires that we draw on multiple disciplines. For the iCOACH team, we required clarification of conceptual linkages in order to: *1) ensure that data collection at the case sites efficiently captures important variables across levels*; and *2) guide data analysis across perspectives and disciplinary approaches*. Concept mapping [24] offers a useful approach to identify how seemingly disparate theoretical frameworks can be brought together in a single study. The **aim** of the paper is to link concepts across different theoretical framework to guide the analysis in “whole systems” on integrated care for complex patients. As such, we used a concept mapping method to answer the research question: *How are concepts across different theoretical frameworks used to guide the iCOACH study linked?*

## Theoretical approaches used by the iCOACH team

### Team structure

The iCOACH research team is organized into four teams (patient/carer, provider, organization, and policy) comprised of both Canadian and New Zealand researchers. Each brought forward a disciplinary perspective and theoretical lens with which to study implementation of ICBPHC models. Each team developed a protocol for data collection that would capture issues pertinent to their level of analysis. While four protocols were then generated, data collection at case sites occurred concurrently, requiring careful consideration of how protocols overlapped in terms of the data collection and analysis. This section provides an overview of the theoretical approach used by each team.

### Patient/carer research team

The patient/carer research team sought to understand ICBPHC from the perspective of the patients and carers being served by the model. The team adopted Ham’s ***Ten Characteristics of the High-Performing Chronic Care System framework*** [15]; a heuristic model developed to identify factors enabling the implementation of the CCM. The model was developed through an exploration of the evidence on implementation of the CCM, and includes a range of factors that relate to access, disease management, and service design and delivery.

To capture key elements of the Ham’s framework and the CCM related to the patient and carer perspectives, the Patient Assessment of Chronic Illness Care (PACIC) instrument [21] was adopted, and supplemented by instruments that enquired in more depth about financial deprivation and holistic assessment of health outcomes from health care, including physical, mental, and spiritual relationships (related to patient and carer demographics). Key elements from Ham’s framework related to the patient and carer perspective captured using the PACIC include:

1. *Self-management and prevention*: Patient’s perspective on their role in their health care and whether they have adequate supports to self-manage. Also related to chronic disease management and illness prevention activities.
2. *Care coordination*: Patient perspective on alignment of care across different providers.
3. *Access to services (and types of services delivered)*: Patient’s perceived ability to get support in accessing needed services in the clinic, by specialists and services in the community.

### The provider research team

The provider research team was also guided by Ham’s framework in their approach to understanding implementation of ICBPHC. In order to collect key variables from this framework pertinent to the provider perspective, the team used the *Assessment of Chronic Illness Care* (ACIC) tool, a survey designed to capture key components of the CCM [22]. Domains from Ham’s framework were thus captured along the lines of the 6 key components of the ACIC tool [23] including:

1. *Organization of delivery system*: Orientation of the health system in relation to delivery of chronic illness care.
2. *Self-management support*: Empowering and preparing patients to manage their health and health care through emphasizing the patient’s central role in managing their health, use of effective self-management support strategies that include assessment, goal-setting, action planning, problem-solving and follow-up, and organizing internal and community resources to provide ongoing self-management support to patients.
3. *Community linkages*: Mobilizing community resources to meet the needs of patients. Includes activities like encouraging patients to participate in community program, forming partnerships with community organizations to support and develop interventions that fill gaps in needed services, and advocating for policies to improve patient care [2].
4. *Decision support*: Promoting clinical care that is consistent with scientific evidence and patient preferences. Can include embedding evidence-based guidelines into daily clinical practice, sharing evidence-based guidelines and information with patients to encourage their participation, using proven provider education methods, and integrating specialist expertise and primary care.
5. *Delivery system design*: Organizational practice in the provision of care including aspects of team functioning, team leadership, clinic processes (appointments, follow-up, planned visits), and continuity of care.
6. *Clinical information systems*: Organizing patient and population data to facilitate efficient and effective care, including sharing information with patients and providers in support of care coordination [2].

### The organization research team

The organization research team adopted the ***Organizational Context and Capabilities for Integrating Care (CCIC) Framework [16]*** to guide data collection and analysis. The CCIC Framework is a theoretically grounded model that captures key organizational factors that influence successful implementation of integrated models of care. The Framework includes 17 factors (or concepts) grouped into three categories:

1. *Basic structures of the organization*: Including physical features, resources, accountability mechanisms, IT, and organizational/network design.
2. *People and values associated with the organization*: Including leadership approach, clinician engagement and leadership, organizational/network culture, focus on patient-centredness and engagement, commitment to learning, work environment, and readiness for change.
3. *Key organizational processes*: Including partnerships, delivering care, measurement of performance, and quality improvement practices.

The Framework includes attention to how the external environment (such as characteristics of the intervention and target patient population) may influence each factor, as well as proximal and distal outcomes likely to be related to adopting models of integrated care.

### The policy research team

The policy research team used the ***Health Policy Monitor (HPM)*** [17] survey methodology to explore the role of health and social policy in the implementation of ICBPHC. The HPM is designed to enable comparative policy analysis, capturing information regarding current and historical health policies and reform efforts, policy ideas, as well as change management activities and processes. The methodology focuses on policy actors who have been integral to policy reform and change efforts. The questionnaire is intended to be used to focus on particular policy developments, in this case, the policy developments around ICBPHC.

The HPM questionnaire is organized on a two-dimensional matrix capturing 15 issue categories (matrix 1) as they develop over 7 process stages (matrix 2). It aims to capture and categorize the issue being assessed as well as its implementation. Rather than adopting the questionnaire directly, it is used to guide development of semi-structured interview guides which are organized to capture data at three system levels: ***case site*** (may be within a single organization or set of organizations that constitute a loosely aligned network), ***service matrix (network)*** (organizations or networks like regional health boards that influence how services are delivered, funded and governed), and ***policy subsystem*** (referring to higher system level oversight which may include state as well as non-state actors such as Ministries of Health and accreditation bodies).

Each of the three levels of analysis includes key domains or questions that are explored. At the case site level, four areas of inquiry are explored through directed questions including: 1) *Why the model of care is delivered*; 2) *what services are being provided*; 3) *who is being targeted by the model of care*; and, 4) *how services are delivered*. At the service matrix (or network) key aspects of *delivery arrangements, financial arrangements* and *governance/accountability* structures are captured. Finally, *financial arrangements* and *governance/accountability* structures that occur at the policy subsystem level are examined in this approach.

### Identifying the linkages: A game of 3-D chess

The overlap between disciplinary and theoretical approaches can be understood much like a game of 3-D chess. In this particular game, we have four boards at different levels, each consisting of multiple squares (or concepts). While some squares remain stable at their level, others squares allow our game pieces to jump between levels, creating a series of conceptual trap-doors that connect two or more boards together. By developing a clear understanding of the boards *and* trap-doors, we can comprehensively capture all factors relevant to ICBPHC from a multidisciplinary, whole system perspective. We adopted a concept mapping approach to both locate and understand the nature of these trap-doors.

## Methods

Concept mapping was initially developed by Joseph D. Novak [24] to graphically represent the relationships between concepts using labels, nodes, boxes and links [25,26,27]. Concept maps were originally designed to support learning for school-aged children but have increasingly been used as a tool in research, supporting data collection and analysis [25,26,27,28]. This newer use of concept mapping is well aligned with the aims of our study to guide data collection and analysis while ensuring all critical concepts are captured.

Trochim [28] suggests the concept mapping process can be viewed as an “artistic procedure which yields interpretable, suggestive conceptual pictures and a scientific one based upon sound evidence regarding its validity, reliability, and theory-enhancing value” (p. 87), and further suggests that concept maps provide “suggestive devices, useful for their stimulative or creative value” (p. 88). Here we adopt the “hard art” approach to concept mapping suggested by Trochim, whereby we use rigorous qualitative methods to develop a map to inform both data collection and analysis for the iCOACH study. To this end we adopt two key methods. First is the generation of a concept map to align the four disciplinary approaches and theoretical models used to guide the iCOACH study. Second is a preliminary exploration of one key overlap point using data from one Ontario case site. We focus on a single overlap point, care coordination, to act as an initial “test” of the map and to demonstrate its utility. Further testing of the full map will be done by the research team as we move forward with our data analysis.

### Concept mapping of perspectives on integrated community based primary health care

Novak and Cañas suggest a six-step approach to concept mapping: 1) clearly defining the focus/question; 2) identification of relevant concepts; 3) construction of a preliminary map; 4) map revision; 5) identifying cross-links; and 6) map revision after links identified [26]. In this section we describe the activities of our research team aligned with each of the 6 steps. The iterative analysis approach conducted by multiple members of the research team supports credibility and dependability of the initial map [29] through crystallization of multiple viewpoints (see [30] on crystallization as a method to enhance credibility in qualitative research).

Step 1: Defining the focus/question

The focus for the iCOACH project is to explore implementation of ICBPHC systems, and the question we are aiming to answer with the concept map is *what macro, meso and micro level factors are relevant to implementing these models?* The aim then for this concept map is to produce a descriptive guide for our data collection and analysis approach.

Step 2: Identification of relevant concepts

Each of the four research teams identified relevant theories and tools that point to key concepts to be understood at each level. The previous section describes the theoretical frameworks and approaches identified by each team.

Step 3: Constructing the preliminary map

The following process was followed to construct the preliminary map. Prior to constructing the map, the process described below was introduced in a team meeting and agreed upon by the research team:

1. The lead author initially looked over the concepts within each of the theoretical frameworks and identified conceptual similarity across models. This analysis involved both looking at the description of concepts from the original theoretical models, along with an examination of the operationalization of those concepts in the form of data collection tools (e.g. survey and interview questions, document collection) as well as the analysis strategies set out by the research teams in the iCOACH protocol.
2. Four concept tables were created (one for each team) which linked to theoretical framework and data collection methods.
3. Concept tables were sent to iCOACH team leads (and other team members) for feedback to ensure tables adequately reflected theoretical frameworks. Concept tables were reviewed by 8 team members. Meetings were held with each team lead to discuss the tables and approach. The types of feedback provided varied by team. In some cases, research team leads (and/or other members) reviewed the table to check that concepts and items were adequately reflected. Other team leads provided broader feedback with regard to their overall approach, conceptual framework, and anticipated methodology.
4. Based on this feedback, the concept tables, and protocols, conceptual overlaps between team protocols were identified. Concepts were grouped when they: 1) directly overlapped in terms of what was being captured; and/or 2) were fully or partially conceptually related (e.g. Patient characteristics are related to “who” the ICBPHC project is targeting). Table 1 overviews concept groupings.

**Table 1:** Concept overlap across four levels: trap doors.

Concept groups	Analytic level	Concept	Definition

System structure and design	Policy	Policy subsystem – funding and governance	Applicable legislation, regulation, guidelines. Eligibility criteria for patients to enter the program.
	Patient-carer	Access to services (universal coverage)	Patients have access to all basic necessary health care services when needed which are free at the point of care. Related to system level concept of university coverage captured in the Ham model.

System governance	Policy	Funding and governance arrangement – all levels	How services are delivered in terms of governance structures and payment mechanisms.
	Organization	Organizational infrastructure and design: Resources and Governance	Resources: Amount and sources of funding Governance: Board composition, activities and oversight.

Organization structure and leadership	Organization	Leadership, strategy and clinician engagement	Leadership style of the organization and level to which clinicians and frontline providers are engaged in decision-making (e.g. centralized or distributed/shared, responsive approaches; receptivity to new ideas).
	Provider	Organization of delivery system – leadership	Elements of the organization of the delivery system specifically focused on leadership and support for frontline providers.

IT systems design and utilization	Organization	Organizational infrastructure and design: IT systems	Information technology systems available in and across organizations (e.g. decision support tools, electronic information systems, electronic health records, interoperability).
	Provider	Provider use of IT system	What information technology systems (in particular clinical information systems) are used by providers in clinical practice.

System level values and perspectives	Policy	Why deliver this model of care – case site level	The organizational impetus for delivering this particular model of care and the nature of the care provided.
	Organization	Social-Psychological context: Organizational culture	The overarching focus and goals of the organization, as well as the character of the organization, managerial style and employee support and overall cohesion. Includes organizational climate concepts such as openness and flexibility in the organization, change readiness, and employee burnout/stress.

Care Coordination	Policy	Delivery arrangements – case site and matrix level	Who delivers care in terms of direct care delivery (in-house staff), contracting out, partnerships, and/or referrals.
		How services are delivered – case site and matrix level	The extent to which models use integrating/coordinating mechanisms (e.g. inter-disciplinary teams, integrated care plans, information technology used to integrate).
	Organization	Teamwork and collaboration	Use of multiple modes of communication within and between teams, feelings of teamwork, cooperation, information sharing and goal alignment, staff input in decision-making.
		Inter-organizational linkages	Formal and informal connections made between different organizations in a network. Including connections between different teams. Focus on process of working across organizational boundaries.
	Provider	Delivery system design	Team level functioning and leadership and continuity of care.
		Community linkages	Providers ability to link patients to outside resources and partnerships with community organizations.
	Patient-carer	Care coordination	Patient and carer perceptions of level to which their care from different providers is aligned.

Person-centred design	Policy	Who is targeted – case site level	Which individuals and communities are being served by the project defined in terms of their acute and chronic illness profile, their geography, culture or needs.
	Provider	Delivery system design – population management	Adopting a systematic approach to population management, and seeking ways to meet community needs.
	Patient-carer	Patient and carer demographics	Information regarding patient and carer personal information (i.e. gender/sex, age), health and social status, culture and ethnicity, beliefs and values.

Program design and monitoring	Policy	What services are delivered – case site level	What types of services are provided by the project.
		Governance structure – matrix and subsystem levels	How agencies fund and oversee projects (governance and accountability arrangements).
	Organization	Clinical processes	Use of clinical practice guidelines (adoption, availability and organizational support for adoption.
		Continuous quality improvement (CQI)	Adoption of quality improvement processes (strategies, committees, patient experience and outcome collection measures).
	Provider	Types of services	Services delivered to patients and quality improvement processes.
		Self-management support	Services specifically around supporting self-management, assessment and documentation.
		Decision-support	Use and availability of decision-support tools, enabling use of evidence based practice.
	Patient-carer	Types of services used	Services used by both patients and carers.
		Self-management and prevention.	Reported self-management support received, as well as support for patients to engage in their care and do more self-care activities, such as engaging in goal-setting.


Step 4: Revising the map

An initial map was constructed which grouped concepts together in boxes rather than linking using lines. This initial map was presented to the full iCOACH team at a team meeting where it was discussed and modified based on team feedback (approximately 20 members of the research team were in attendance). CMapTools software was then used to visually depict the revised concept map.

Step 5: Seeking cross-links

A sub-group of the broader iCOACH team (the co-authors on this paper) was brought together to identify cross-links, do a final revision, and publish findings. Co-authors reviewed and revised the map, and agreed upon preliminary cross-links for the map. We agreed as a team that the links named in the map would need to be tested and validated using data from the iCOACH study.

Step 6: Revising the map

We used CMapTools to add in cross-links agreed upon in step 5. This version was reviewed again by the co-authors of this paper and consensus was reached with regard to the visual map and content.

## Results

### Concept map

**Figure 1:**
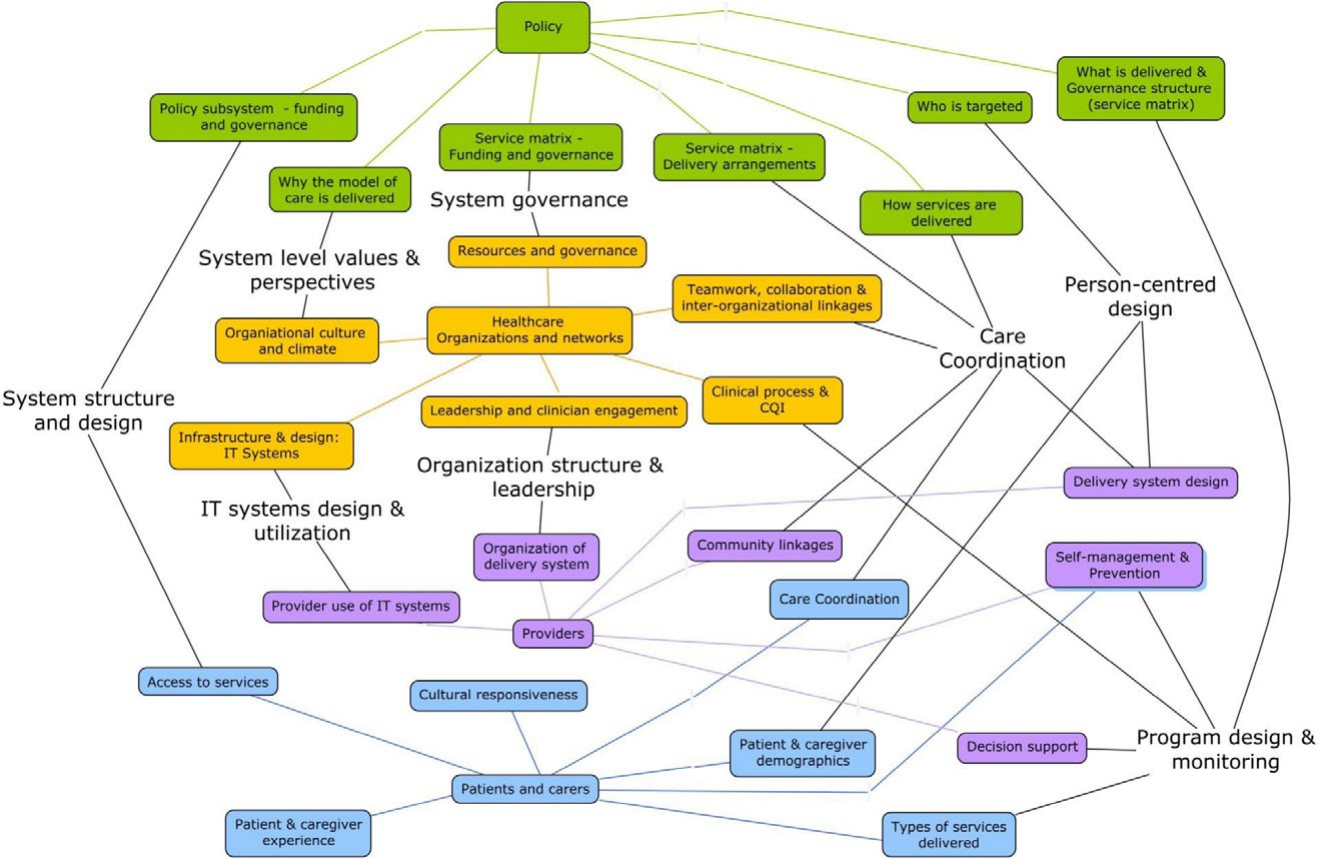
Implementation of Integrated Community-Based Primary Health Care Concept Map.

Figure 1 visually presents the concept map. Each analytic level is represented down the center of the map and is given a unique colour: policy level is green, organization level (written as healthcare organizations and networks) is orange, provider level is purple, and patient/carer level is blue. The central boxes of each level are linked to their related concepts in the same coloured boxes via similarly coloured lines.

Conceptual overlap across levels is depicted through black lines linking concepts from different levels. As noted in the methods section these concepts are similarly defined and operationalized (e.g. similar survey and/or interview questions are used to capture the concept), however the nature of their relationship in practice is yet to be determined. To help us make sense of the map we include labels where concepts intersect that relate to what we see as 8 overarching concepts:

1. System structure and design
2. System governance
3. Organization structure and leadership
4. IT systems design and utilization
5. System level values and perspectives
6. Care coordination
7. Person-centred design
8. Program design and monitoring

We acknowledge that this is a preliminary attempt to categorize concept groups which will need to be explored through the process of data analysis. A full exploration of these overlaps is beyond the scope of this paper and will be conducted through analysis of iCOACH data over the course of future papers.

### Exploring interconnections: the example of care coordination

To demonstrate the utility of the concept map, data from the iCOACH study was used to explore the ***care coordination*** theme and the related concepts across the four perspectives. Care coordination is a critical component in the integration of services [31] and as such is a useful place to start our analysis. One case from Ontario was selected to extract findings. Transcribed interviews from policy makers, organizational managers, front-line providers and patients and carers in each jurisdiction had been coded as part of the first stage of analysis along common coding structures specific to each level of analysis. This initial coding process was conducted by different members of the iCOACH research team from Ontario, Quebec and New Zealand through a multi-phased iterative approach in which coding was validated through multiple rounds of double coding, discussion, and consensus building as is consistent with qualitative analysis methods used to guide analysis of the iCOACH study [32,33]. This approach created a set of rigorously coded data, allowing for the team to easily extract data on similar concepts across cases and jurisdictions. Coded data that related to overlapping concepts related to care coordination were extracted from the one Ontario case for the analysis presented in this paper.

Care coordination is a widely explored concept with multiple theoretical understandings and activities described in the literature. The Agency for Healthcare Research and Quality in the United States has defined care coordination as:

“… the deliberate organization of patient care activities between two or more participants (including the patient) involved in a patient’s care to facilitate the appropriate delivery of health care services. Organizing care involves the marshaling of personnel and other resources needed to carry out all required patient care activities and is often managed by the exchange of information among participants responsible for different aspects of care.” [34] (p. 6)

In the concept map, we see an important grouping of concepts around care coordination. While care coordination is explicitly included in the patient/carer theoretical enquiries, it is not explicitly written into the frameworks used by the provider, organizational and policy research teams. The concept map offers insight into where care coordination fits into other theoretical perspectives, and where we might see differing conceptualizations and approaches to this concept. Similarities in concepts arising from different theoretical approaches and levels of analysis is a key advantage of using a concept map approach, allowing for a multidisciplinary analytic approach which recognizes multiple definitions and conceptualizations of similar concepts.

From the policy perspective, *how services are delivered* and *delivery arrangements* are concepts used by the research team to capture the extent to which studied models use integrating or coordinating mechanisms. While this is primarily a descriptive concept, the policy perspective approach suggests we explore historical trends, and related policy subsystems relative to how services are delivered. Current and historical policies that shape the system and health care institutions that can create barriers or enablers to *how services are delivered* are included in this concept from a policy perspective.

From the organizational perspective the map shows how *inter-organizational linkages, collaboration* and *teamwork* are linked to the concept of care coordination. The connection here is a bit more obvious as inter-organizational linkages and partnerships create processes such as sharing administrative and clinical information, referral systems, and joint accountabilities that are important to cross-organizational coordination of care [16]. Similarly, at the provider level, *community linkages* between individual providers can enable coordination of patient care across health and social care boundaries [35]. *Delivery system design* specifically around team functioning, leadership and continuity are additionally key components of care coordination at the provider level.

### Care Coordination in Ontario

Elsewhere in this special issue Breton et al. [19] offer detailed descriptions of case examples from Ontario and Quebec in Canada, and New Zealand. The Ontario case of focus for the analysis presented in this paper is the Integrated Client Care Program, a collaborative model of care based on a partnership between a Family Health Team and Community Care Access Centre located in Toronto with strong partnerships with a local hospital and other community agencies. This program focuses on high needs older adults with the aim to coordinate care delivery across the multiple providers in primary, community and acute care settings.

#### The policy perspective on care coordination: How services are delivered and delivery arrangements

Organizational managers from the Integrated Client Care Program identify significant system fragmentation which results in difficulties when different sectors (e.g. primary, acute and community care) seek to coordinate patient care. One hospital manager clearly defines the issue of fragmentation with regard to patients with complex care needs transitioning from hospital to home:

“When the reality is [the hospital], maybe, see that patient for like 1% of their health care journey and the majority of the care that they receive is actually in primary care. And yet, primary care often is the one sector that doesn’t get engaged in the delivery, or in, actually, in defining what the care’s going to look like for that patient post discharge from the hospital. And because primary care is not engaged in that ward, that patient often […] fall through the gaps all the time.” (SE-05 – Hospital manager)

This sentiment of fragmentation is echoed by a policy-level informant from a CBPHC partner who identifies that while excellent care delivery occurs, it does so in silos:

“We have pockets of excellent primary care and pockets of excellent homecare. They almost never integrate […] So I think that is the vision is to integrate them.” (M5)

In the Ontario example, new legislation, *The Excellent Care for All Act*, 2010 [36], establishes quality reporting and delivery process requirements for health care providers to enable patient-centred care delivery, which is funded and governed through regional bodies (at the service matrix level). A manager/clinician from the Family Health Team in this case describes how legislation provides an opportunity to shift how services are delivered through development of a new program that could work to improve integration:

“… the good luck of the Excellent Care for All Act really coming right at the time when we’re thinking about improving care for patients and making patients the centre of care. So that was exactly what the Virtual Ward was, um, intending to do. And so a major healthcare policy shift allowed us to really have the driver, um – it’s, you know, at hand to also bring CCAC onboard, because it aligned, um, with, um, major healthcare policies.” (SE-13 – Family Health Team manager/clinician)

#### The organizational perspective of care coordination: Organizational linkages and teamwork

In seeking to adopt new programs to address fragmentation and improve coordination, managers seek to develop new inter-organizational linkages and teams. Having a shared vision of integration between organizations as well as clear strategies related to collaboration and inter-organizational linkages are identified as key enablers to building connections. A manager from the Community Care Access Centres (CCACs) in the Ontario case reflects:

“We have one strategy across the province for all CCACs around building meaningful relationships with primary care and building integrated teams in local geographies across the province. One such example is with the [family health team].” (SE-14 – CCAC manager)

Beyond just the strategy and vision of collaboration, organizational managers from the case identify the importance of having a culture of teamwork to develop organizational linkages and support integrated care delivery:

“Um, the teamwork and the organizational culture are interesting because, um, for example in my team, it was really the team itself. I was somehow very lucky to have just wonderful, wonderful team players. Um, and so that set the culture, because you know, it wasn’t that it was just simply my family health team’s culture or CCAC’s culture, or [the hospital’s] organizational culture. But it was the team that established it as a culture that we are all committed to that same outcome and vision.” (SE-13 –Family Health Team manager/clinician)

#### The provider perspective of care coordination: Delivery system design and establishing community linkages

The culture of teamwork and organizational strategy aimed at developing relationships creates an environment to support the development of community linkages and inter-professional teams. One physician reflects on how coming together on patient care serves to solidify relationships with community partners:

“I think it was just, you know, through providers that have provided care for mutual patients that that’s sort of how the relationships have formed. They’ve matured into sort of more collaborative groups. I think they started informally and now have sort of formalized.” (SE-01 – Family Health Team clinician)

Built relationships and a shared culture of collaboration across organizations creates a supportive environment that allows inter-professional teams to communicate and work together to establish processes to coordinate care. Information sharing processes and integrated systems, as well as activities like case conferences allow Family Health Team providers to work closely with other home and community care providers. Another Family Health Team clinician in an allied health discipline describes how case conferences are a particularly helpful process:

“So, it’s kind of like a lot of back and forth. Which can just be time consuming and a little bit frustrating, but rather have everybody at the table, hear the patient’s concerns, come up with a plan together – that way everybody’s sort of the same page rather than the back and forth. I don’t know, I think that’s been helpful.” (SE-02 – Family Health Team clinician)

#### The patient and carer perspective of care coordination: Perceiving coordinated care

Patients and carers want to be confident they are referred for expert care and advice when needed, that the information they have provided to one party is passed on as appropriate to others responsible for components of their care, that they are listened to, respected, and provided with the information and resources they need to manage their conditions. The inter-professional work facilitated through the Integrated Client Care Program seems to provide an integrated experience for patients and carers in that the whole team is aware of the patient needs. As one carer reflects:

“So there’s always (sighs), they’re always there, and they all seem to know what’s going on with Jim, and they come when they’re needed.” (SCa-02 – carer)

#### A whole system perspective of care coordination in the Integrated Client Care Program in Ontario

Using the concept map to approach analysis we can see how different disciplinary lenses assign relevance to different aspects of a similar concept. In the above example from Ontario, patients and carers identify that a key aspect of care coordination for them is that they have a team of health care providers who are available to meet their needs when required. For providers, care coordination is about building relationships in teams that exist within and across different organizations. The data presented above would suggest that managers and policy makers see care coordination as being more about creating linkages and addressing systems gaps that could *enable* care coordination at a clinical level.

These different meanings of care coordination for different actors suggest there are multiple ways to enable and measure care coordination. In terms of measurement, if we were to simply count the number of organizational linkages that occurred since adoption of new policy, we may miss out on whether these linkages have come along with development of strong relationships and trust needed to ensure functioning of teams from a provider perspective. We may also miss whether these new linkages are in fact helping meet needs of patients and carers, or whether these add to the confusion of where services can be accessed. Similarly, different meanings of care coordination suggest there are likely different drivers as well. Where strong relationships and teamwork are important to providers, aligned vision and shared culture is more important to create linkages between organizations.

These differing perspectives are not issues of semantics. Rather, they identify a key point in the implementation process of integrated models of care. The critical issue is about perspective. Starting at the perspective of any one of these four levels will lead to a different design response to the issue of ‘care coordination’. This highlights the importance of developing a “whole system” framework that can work across the four levels of patient/carer, provider, organization, and policy.

Diving more deeply into these trap-doors is beyond the scope of this paper. This preliminary analysis does, however, demonstrate how a multidisciplinary approach requires attention to new, potentially unexpected areas of inquiry that can better inform implementation of ICPBHC.

## Discussion

Using a concept map to guide multidisciplinary and cross-level analysis demonstrates the value that comes from linking across levels to better understand integrated models of care, and how different perspectives understand and assign value to similar ideas. While concepts from different theoretical perspectives may be similar in definition, the cross-level analysis shows how looking at these concepts through different perspectives reveals different realities which can be brought into focus.

The analysis guided by the concept map points to three important dimensions of any concept which requires our attention. First, *what is the meaning of the concept from different perspectives*? In the Ontario case example, it is found that what care coordination means to the patient is very different then what it means to health care organization managers and policy makers. Second, *how do different actors value and measure the concept?* How does a policy maker know care coordination has been achieved? When does a provider feel like the care they deliver is coordinated across their inter-professional team? And finally, *what are the perceived drivers of the concept across levels?*

This last point regarding drivers of different concepts is particularly important in our understanding of implementation of ICBPHC. In the Ontario example we see how what is important to providers is having strong relationships with inter-disciplinary team members, supported by agreed roles, responsibilities, boundaries, funding and reporting, in order to coordinate care delivery. For policy-makers, however, the interest is in achieving high-quality care delivery as captured through performance indicators. The risk is in the implementation of these activities. Relationship development in inter-professional teams “requires time, interaction, and focused attention” [37, p. 231], which can potentially take time away from direct patient care. Performance indicators in primary care often focus on access time for patients [38], leaving little room for providers to work on team and relationship building outside of patient visits. Other conflicts may arise for organizations who seek to develop new partnerships within a system that includes competitive procurement. Previous studies have shown that competitive procurement processes (intended to meet policy objectives of quality and efficiency) can act as a significant barrier to partnership [39].

### Limitations

What is presented in this paper is a methodological approach to multidisciplinary whole systems studies using concept mapping. The emphasis in the presented work is on the development of the map with only an initial look into analysis. An important limitation here is that the analysis of the Ontario case with regard to care coordination is a preliminary exploration. Future steps in this work will require a more in-depth study of conceptual overlap of care coordination, as well as the other 7 conceptual areas of the map using cross-disciplinary theories to tease out some of the questions that have been posed by this work. Additional testing of the map using empirical data will enhance credibility and dependability of the map. The map should further be validated through empirical testing. Future analysis, much of which is currently underway, will require the work of multidisciplinary research teams working across different perspectives to adequately tease apart meanings, measures and drivers of shared concepts.

## Conclusion

The concept map and analysis presented in this paper marks an important first and foundational step towards a more in-depth cross-disciplinary analysis of the integrated models of CBPHC to be conducted as part of the iCOACH study. The map in its current form requires a clearer understanding of exactly how concepts are related in practice. We present an initial exploration of these relationships, identifying three dimensions of analysis that could be adopted in future explorations of the map using data from the iCOACH study. The methods and preliminary analysis presented in this paper can be used as a guide by other multidisciplinary research teams exploring implementation of integrated models of CBPHC and/or who are using multiple theoretical models to engage in “whole systems” health services research. The collaborative nature of iCOACH across New Zealand and Canada illustrates that the approach is also a viable strategy for alignment and comparison of international models of service delivery. The concept map should be viewed as a living document, one that grows and develops as analysis of data continues. Importantly, identifying which concepts may be more important to the implementation of ICBPHC models should be explored with particular attention to the drivers of similar concepts for different stakeholders across the system.
